# Learning from the COVID-19 pandemic: health care disturbances and telemedicine as an alternative rheumatology practice in Indonesia

**DOI:** 10.1186/s12913-023-09389-5

**Published:** 2023-05-08

**Authors:** Faisal Parlindungan, Sumariyono Sumariyono, Rudy Hidayat, Suryo Anggoro Kusumo Wibowo, Anna Ariane, Johanda Damanik, Abirianty Priandani Araminta, Khadijah Cahya Yunita

**Affiliations:** 1 Rheumatology Division, Department of Internal Medicine, Faculty of Medicine, University of Indonesia, Jakarta, Indonesia; 2 Department of Internal Medicine, University of Indonesia Hospital, Depok, Indonesia; 3 Rheumatology Division, Department of Internal Medicine, Dr. Cipto Mangunkusumo National General Hospital, Jakarta, Indonesia; 4 KRT Setjonegoro General Hospital, Wonosobo, Indonesia

**Keywords:** Health services, Health behavior, Rheumatology, Telemedicine, COVID-19

## Abstract

**Background:**

Coronavirus disease 2019 (COVID-19) affects health care services. Our aim was to assess health care disruptions, treatment interruptions, and telemedicine reception regarding autoimmune rheumatic diseases (ARDs) in Indonesia.

**Method:**

A cross-sectional population online-based questionnaire was conducted in Indonesia from September to December 2021.

**Results:**

A total of 311 ARD patients were included, of whom 81 (26.0%) underwent consultations via telemedicine during the COVID-19 pandemic. The respondents showed increased concern about their susceptibility to COVID-19 (score of 3.9/5). Approximately 81 (26.0%) avoided hospital visits, and 76 (24.4%) stopped taking the medication without medical advice. Respondents’ concerns correlated with their social distancing behaviors (p value 0.000, r 0.458). Respondent concerns, behaviors, and blocked access to the hospital during the pandemic were associated with avoiding hospital visits (p value 0.014; 0.001; 0.045; 0.008). Sex was associated with stopping medication (p value 0.005). In multivariate analysis, blocked access and sex remained significant. Approximately 81 (26%) respondents who used telemedicine services during the COVID-19 pandemic as an alternative medical consultation method showed high satisfaction (3.8/5).

**Conclusion:**

Health care disruptions and treatment interruptions were affected by patients’ internal and external factors during the COVID-19 pandemic. Telemedicine may be the best option to address barriers to health care access in Indonesia’s rheumatology practice during and after the pandemic situation.

**Supplementary Information:**

The online version contains supplementary material available at 10.1186/s12913-023-09389-5.

## Introduction

Coronavirus disease 2019 (COVID-19) was first identified in December 2019. Due to the highly pathogenic and rapid spread of SARS-CoV-2 worldwide, the World Health Organization (WHO) declared COVID-19 a global public health concern. To control the spread, countries, including Indonesia, implemented health protocols to regulate social distancing behaviors and restrict nonvital social mobility, such as public transportation and traveling across regions or borders [1–3].

Besides, autoimmune rheumatic disease (ARD) patients are at risk of being infected and may have a poorer outcome of SARS-CoV-2 infection due to immune dysregulation and immunosuppressive effects of ARD treatment, [4, 5] which increases patients’ concerns about being infected with COVID-19 and promote preventive social distancing behavior [6, 7]. Moreover, the lockdown policy was a barrier to access for patients. These problems became a challenge for ARD patients in accessing continuing medication and disease monitoring to prevent the progression of the disease and worse outcomes [6–11]. Thus, this study provided new insight into factors that affected health care disruptions and treatment interruptions.

Alternatively, the WHO advised switching to telemedicine during the pandemic [12, 13]. Moreover, the Asia-Pacific League of Associations for Rheumatology (APLAR) published recommendations for telemedicine in rheumatology practice in early 2022, [14]. in addition to the Indonesian government telemedicine guideline during the pandemic [15, 16]. Prior to the pandemic, telemedicine was used to provide teleconsultation across regions, nations, and even international space stations. In rheumatology, telemedicine has been used to monitor chronic autoimmune diseases and even deliver biological agents [17–19]. Hence, we evaluated patients’ telemedicine reception as an alternative in health care practice in the pandemic situation in Indonesia.

Currently, in 2023, the COVID-19 pandemic status shifted into an endemic status, and lockdown policies are gradually being rescinded. However, due to Indonesia’s unique geography and sociodemographic conditions, barriers to access remain a major challenge. Finally, learning from this pandemic, there should be considerable changes in rheumatology practice to meet patients’ needs.

## Subjects and methods

### Study population

A cross-sectional study was conducted in Indonesia using a national online survey (Google Forms). With the support of ARD communities and rheumatologists in several rheumatology centers in Indonesia, we recruited participants to fill out an online submission form to be collected consecutively from September to December 2021.

The inclusion criteria were as follows: (1) respondents had to be at least 18 years of age; (2) respondents who lived in Indonesia during the first and second COVID-19 wave; and (3) respondents who were diagnosed with one or more autoimmune rheumatic disease(es) such as rheumatoid arthritis (RA), systemic lupus erythematosus (SLE), psoriatic arthritis (PsA), ankylosing spondylitis (AS), Sjögren’s syndrome, and scleroderma/systemic sclerosis. The specific criterion for telemedicine was as follows: (1) Respondents who used conventional or hospital telemedicine services during the COVID-19 pandemic. The exclusion criterion was as follows: (1) respondents who did not agree to participate in this study.

### Data collection

The study protocol was provided on the first page of the online questionnaire, and each participant gave consent to continue to participate or decline to participate voluntarily. All responses were anonymous, and each respondent could only complete the questionnaire once. The questionnaire was conducted in Bahasa, Indonesia. The questionnaire was a self-developed questionnaire that was built based on available previous studies using the health belief model and the health-seeking behavior theory (Additional File 1) [6, 20–22]. The collected data were pooled in a Google spreadsheet.

The questionnaire was divided into three sections: the first section evaluated demographics, medical history, and current treatment; the second section evaluated respondents’ concerns, behaviors, and external factors that contributed to health care disruptions; and the third section assessed respondents’ satisfaction with telemedicine as an alternative type of health care consultation during the COVID-19 pandemic.

The questionnaire regarding patient concerns about COVID-19 infection, social distancing behavior, and satisfaction was rated on a five-point Likert scale as follows: “never (1),“ “rarely (2),“ “sometimes” (3), “often (4),“ and “always (5)”. We used the respondent’s district location to determine the level of travel restrictions by the government during the second wave of the COVID-19 pandemic based on the Ministry of Home Affairs’s instructions for the lockdown policy [2, 3]. For the outcome, we designed the questionnaire to assess health care disruptions and treatment interruptions as “yes” or “no” questions. The questionnaire regarding satisfaction with telemedicine was rated on a five-point Likert scale as follows: “very dissatisfied (1),“ “dissatisfied (2),“ “neutral (3),“ “satisfied (4),“ and “very satisfied (5).“

### Statistical analysis

Collected data were analyzed using SPSS 25 (SPSS Inc., Chicago, IL, USA) for Windows 11. Continuous variables are presented as the means (standard deviations) when normally distributed and medians (minimums-maximums) when not normally distributed. Categorical variables are presented as percentages. The chi-square test explored the association between patients’ concerns, social distancing behaviors, and external factors in health care disruptions such as avoid a hospital visit and treatment interruptions such as stopping the medication. All statistical tests were 2-sided, P values less than 0.05 were considered significant, and P values less than 0.001 were considered highly significant.

## Results

### Demographics of the respondents

We excluded three out of 314 recruited respondents because they did not meet the inclusion criteria. The remaining 311 respondents’ demographic and baseline characteristics are summarized in Table 1. The median age of respondents was 40 years (min 21-max 68), and the majority of the respondents were female (299, 96.1%). SLE was the most common ARD in this study (217, 70.1%), followed by RA (86, 27.3%). In addition, 54 (17.4%) respondents had more than one ARD diagnosis. Most of the respondents received nonbiologic disease-modifying antirheumatic drugs (DMARDs) (74.3%) and glucocorticoids (71.4%). Approximately 81 (26%) respondents reported avoiding hospital visits, and 76 (24.4%) respondents had ever stopped ARD medication without medical advice.

**Table 1:** Demographic and Baseline Characteristics of Patients

Characteristic	Population (n = 311)
Age, mean ± SD	40 (21–68)
Female, n (%)	299 (96.1)
Region, n (%)	
Java-Bali	260 (84)
Outside Java-Bali	51 (16)
Region Level of PPKM (2nd Wave, August 2021), n (%)	
1	0 (0)
2	5 (1.6)
3	61 (19.6)
4	245 (78.8)
Rheumatic Condition, n (%)	
Systemic Lupus Erythematosus	217 (70.1)
Rheumatoid Arthritis	86 (27.3)
Systemic Sclerosis/Scleroderma	23 (7.4)
Sjogren Syndrome	20 (6.4)
Ankylosing Spondylitis	7 (2.3)
Psoriatic Arthritis	7 (2.3)
Vasculitis	5 (1.6)
Medications, n (%)	
Non-Biologic DMARD	231 (74.3)
Glucocorticoid	222 (71.4)
Anti-Malaria	63 (20.3)
No medication therapy	9 (2.9)
Biologic Agent	6 (1.9)
Type of Health Care Disruption, n (%)	
Need to avoid a hospital visit	81 (26.0)
Stopped medication without medical advice	76 (24.4)

SD: Standard Deviation; PPKM: DMARD: Disease-Modifying Anti-Rheumatic Drug PPKM (Pemberlakuan Pembatasan Kegiatan Masyarakat/Restriction on Community Activity)

### Factors affecting health care and treatment disruptions during the COVID-19 pandemic

**Fig. 1:**
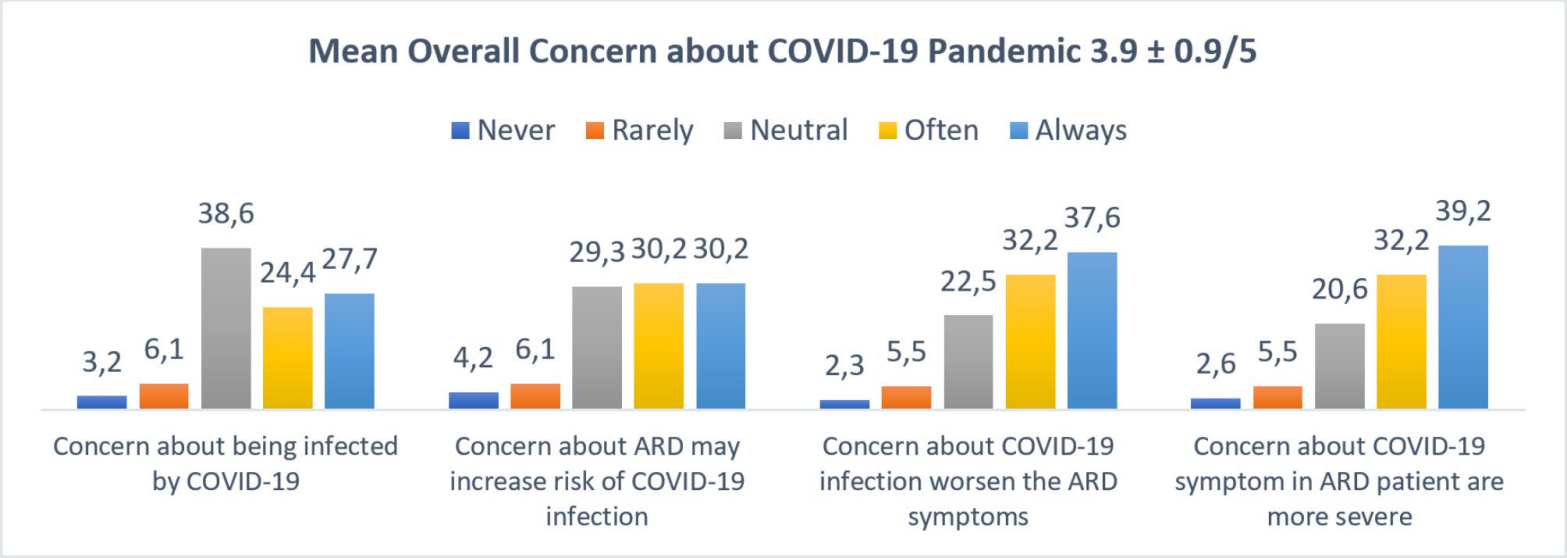
Respondent’s Concern of COVID-19 Pandemic (%)

First, regarding the respondents’ concerns about being infected with COVID-19, the majority were neutral (38,6%), followed by 27.7% who were always concerned and 24.4% who were often concerned about being infected with COVID-19. Regarding basic knowledge about COVID-19 infection, the majority of the respondents were always (30.2%) and often (30.2%) concerned that ARD conditions may increase the risk of COVID-19 infection, followed by those who were neutral (29.3%). Moreover, some respondents were always (37.6%) concerned that COVID-19 disease may worsen their ARD condition, and some were always (39.2%) concerned that COVID-19 symptoms among people with ARDs are more severe than those among healthy people. In addition, as seen in Fig. 1, the mean overall concern score was 3.9 ± 0.9 from the maximum score of 5.

**Fig. 2:**
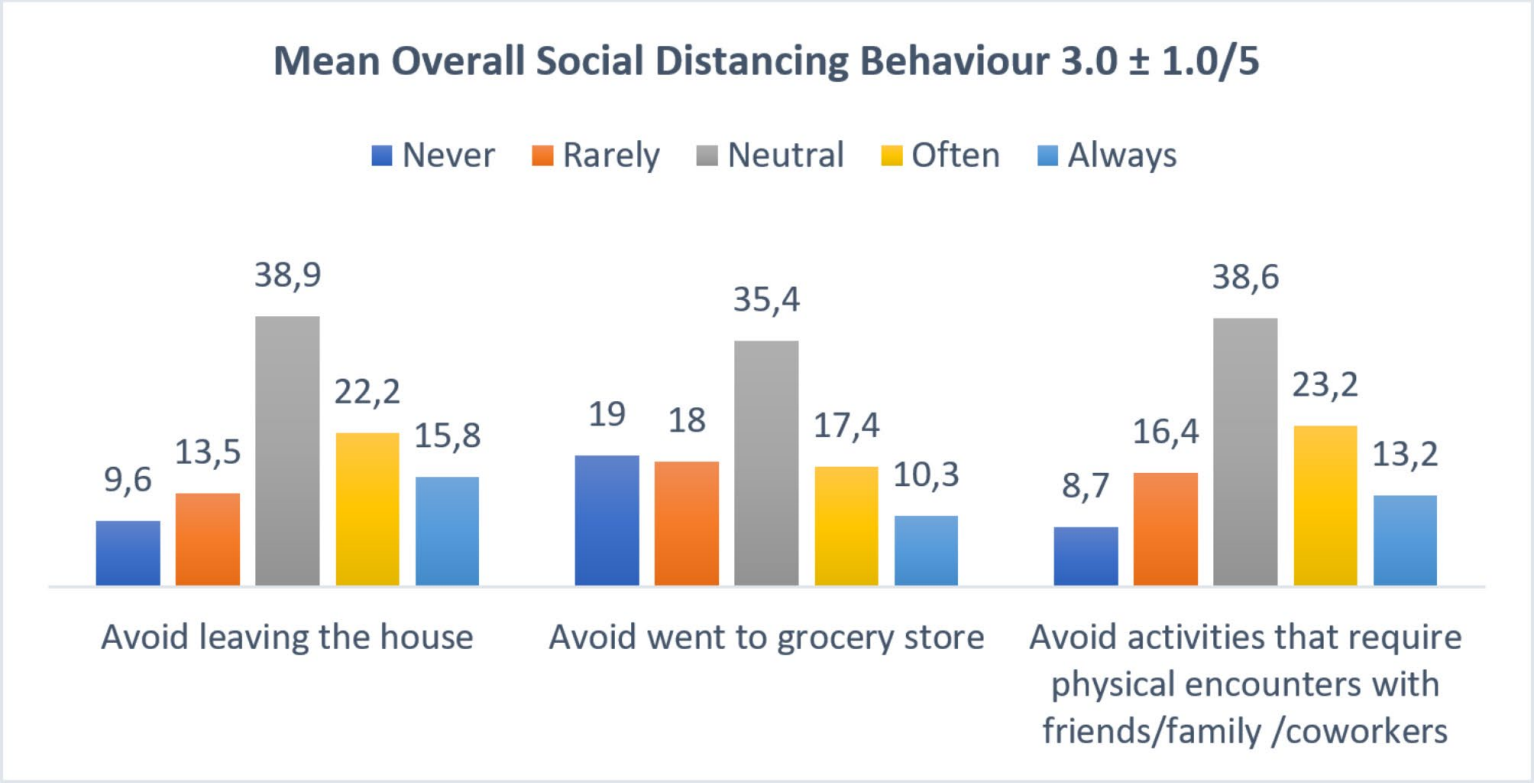
Respondent’s Social Distancing Behavior during COVID-19 Pandemic

**Fig. 3:**
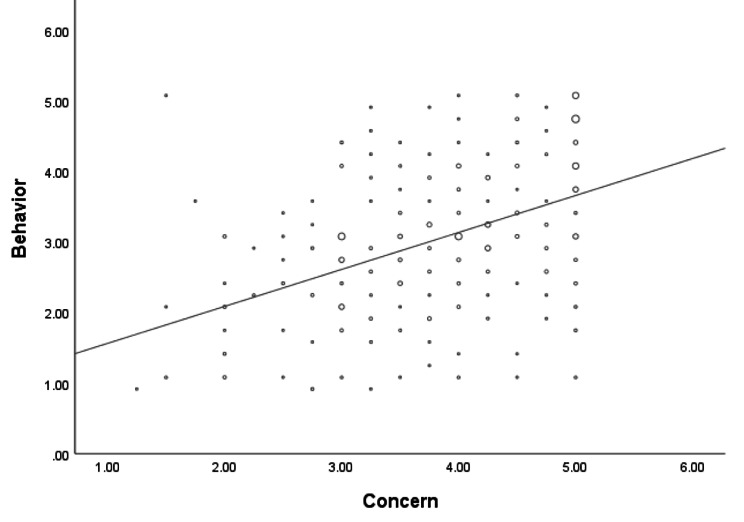
Scatter Plot Correlation between Respondents’ Concern on COVID-19 Infection and Social Distancing Behavior

Second, regarding respondents’ social distancing behaviors during the COVID-19 pandemic, the majority of our respondents sometimes (38.9%) avoided leaving the house, followed by those who often (22.2%) avoided leaving the house. In contrast, 35.4% of our respondents sometimes (35.4%) avoided going to the grocery store, followed by those who never (19%) and rarely (18%) avoided going to the grocery store. Moreover, 38.6% sometimes avoided meeting in person with colleagues and families, followed by those who often (23.2%) avoided meeting in person. As shown in Fig. 2, the mean overall social distancing behavior score was 3.0 ± 1.0 from the maximum score of 5. In addition, as seen in Fig. 3, respondents’ concerns were positively correlated with their social distancing behaviors during the pandemic (p value 0.000, r 0.458).

Third, regarding external factors that contributed to health care disruptions, most of our respondents lived on the Java and Bali islands, where 245 (78.8%) lived in level 4 PPKM regions (Pemberlakuan Pembatasan Kegiatan Masyarakat/Enforcement of Restrictions on Community Activities). PPKM level 4 was the highest level of this community activity restriction policy, and the implication of this was that 240 (77.2%) of our respondents had blocked access to the hospital during the implementation of PPKM. Moreover, the majority of our respondents used private transportation (64.3%) compared to public transportation (35.7%) and both modes of transportation were severely affected by PPKM.

Furthermore, the bivariate analysis of all variables related is shown in Table 2. First, there was a significant association between sex and stopping medication without medical advice (p value 0.005). There was also an association between respondents’ concerns about infection, social distancing behaviors during the pandemic, and blocked access during PPKM (p value 0.014, 0.001, and 0.045).

Finally, multivariate analysis of the respondents’ concerns, behaviors, and blocked access to the hospital is shown in Table 3. A strong association was found between blocked access to the hospital during the pandemic and the need to avoid hospital visits (OR 1.786; CI: 1.008–3.162) and between sex and stopping medication without medical advice (OR: 4.667; CI: 1.436–15.168).

**Table 2:** Factors Associated with Healthcare and Treatment Disruption

Characteristic	Population (n = 311)	Need to Avoid Hospital Visit (p-value)^#^	Stop Medication without Medical Advice (p-value)^#^	The Use of Telemedicine (p-value)^#^
Age, n (%)				
Over 45 years old	87 (28.0)	0.776	0.454	0.616
Below 45 years old	204 (65.6)			
Sex, n (%)				
Female	299 (96.1)	0.209	0.005*	0.054
Male	12 (3.9)			
Education Level, n (%)				
Graduate School	158 (50.8)	0.633	0.918	0.077
Primary-Secondary	153 (49.2)			
Community Member, n (%)				
No	38 (12.2)	0.723	0.604	0.663
Yes	273 (87.8)			
Overall Concern, Mean ± SD/Total Score	3.9 ± 0.9/5			
Poor (≥ 2.5), n (%)	282 (90.7)	0.014*	0.679	0.520
Fine (< 2.5), n (%)	29 (9.3)			
Overall Behavior, Mean ± SD/Total Score	3.1 ± 1.0/5			
Poor (≥ 2.5), n (%)	221 (71.1)	0.001*	0.559	0.121
Fine (< 2.5), n (%)	90 (28.9)			
The region, n (%)				
Non-Java Bali	51 (16.4)	0.549	0.207	0.343
Java-Bali	260 (83.6)			
Level of PPKM, n (%)				
4	301 (96.8)	0.081	0.414	0.181
≤ 3	10 (3.2)			
Blocked Access during PPKM, n (%)				
No	240 (77.2)	0.045*	0.251	0.642
Yes	71 (22.8)			
Transportation, n (%)				
Public	111 (35.7)	0.981	0.810	0.016*
Private	200 (64.3)			

#Chi-square test; * <0.05: significant; ** <0.001: highly significant; SD: Standard Deviation; DMARD: Disease-Modifying Anti-Rheumatic Drug; PPKM (Pemberlakuan Pembatasan Kegiatan Masyarakat/Restriction on Community Activity)

**Table 3:** Factors Affect Healthcare and Treatment Disturbance by Multivariate Logistic Regression

Characteristic	OR	95% CI	p-Value
Need to Avoid Hospital Visit			
Blocked Access to The Hospital	1.786	1.008–3.162	0.047*
Stop Medication without Medical Advice			
Sex	4.667	1.436–15.168	0.010*

*p < 0.05: significant; OR: Odd Ratio; CI: Confidence Interval; PPKM: Pemberlakuan Pembatasan Kegiatan Masyarakat/Restriction on Community Activity

### The use of telemedicine and the respondents’ reception

As shown in Table 4, only 81 of 311 (26.0%) respondents reported that they used telemedicine as an alternative consultation method during the COVID-19 pandemic. Several reasons were recorded among nonusers of telemedicine, and the most common reasons were ‘I don’t know about telemedicine’ (126 (54.8%)), followed by ‘I do not need telemedicine services’ (48 (20.9%)), and ‘No telemedicine service is available’ (27 (11.7%)).

**Table 4:** Telemedicine Use

Parameter	Population
The Use of, n (%)	
Not Using Telemedicine	230 (74.0)
Using telemedicine	81 (26.0)
Reasons for Not Using Telemedicine, n (%), n = 230	
I don’t know about telemedicine	126 (54.8)
I don’t need telemedicine service	48 (20.9)
Service is unavailable in my hospital	27 (11.7)
I doubt the accuracy of the service	15 (6.5)
Health insurance doesn’t cover telemedicine service	8 (3.5)
My medical condition needs hospital visit	4 (1.7)
Absent	2 (0.9)

The respondents’ characteristics regarding telemedicine use are illustrated in Table 5. Among telemedicine users, the mean age was 40 ± 12.8 years, and the majority were female (75, 92.6%). SLE was the most common ARD in this study (57, 70.4%), followed by RA (12, 14.8%), with 14 (17.3%) respondents having more than one ARD diagnosis. In addition, most of the respondents received nonbiologic DMARDs (80.2%), followed by glucocorticoids (77.8%). Furthermore, the most preferred modality among the respondents was text/chat consultations (n = 56, 69.1%), followed by video consultations (n = 44, 54.3%), and 27 (33.3%) preferred to fill out an online form for a direct appointment. Finally, approximately 44 (54.3%) respondents preferred telemedicine during the COVID-19 pandemic, and 33 (40.7%) preferred telemedicine after the COVID-19 pandemic was over. For the last question, the respondents were able to choose more than one answer.

**Table 5:** Demographic and Baseline Characteristics of Patients that Used Telemedicine during COVID − 19 Pandemic

Parameter	Population N = 81
Age, mean ± SD	40 ± 12.8
Female, n (%)	75 (92.6)
Rheumatic Condition, n (%)	
Systemic Lupus Erythematosus	57 (70.4)
Rheumatoid Arthritis	12 (14.8)
Psoriatic Arthritis	1 (1.2)
Ankylosing Spondylitis	3 (3.7)
Sjorgen Syndrome	6 (7.4)
Systemic Sclerosis/Scleroderma	4 (4.9)
Vasculitis	2 (2.5)
Medications, n (%)	
Biologic Agent	3 (3.7)
Non-Biologic DMARD	65 (80.2)
Anti-Malaria	16 (19.8)
Glucocorticoid	63 (77.8)
Preferred modality, n (%)	
Chat/text	56 (69.1)
Video-conference	44 (54.3)
Online Form Face-to-Face Appointment	27 (33.3)
Prefer Using Telemedicine during COVID-19 Pandemic, n (%)	44 (54.3)
Prefer Using Telemedicine after COVID-19 Pandemic, n (%)	33 (40.7)

SD: Standard Deviation; DMARD: Disease-Modifying Anti-Rheumatic Drug

**Fig. 4:**
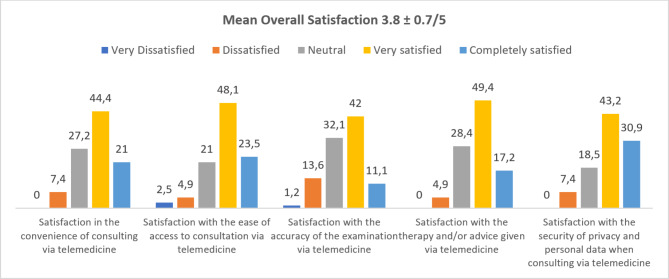
Respondent’s Satisfaction with Telemedicine Uses (%)

Respondent satisfaction with telemedicine services is shown in a bar graph in Fig. 4. The mean overall satisfaction score with telemedicine services was 3.8 ± 0.7, with a maximum score of 5. First, users were very satisfied (44.4%) with the convenience of telemedicine. Second, users were very satisfied (48.1%) with the ease of access to telemedicine. Third, users were very satisfied (42%) with the accuracy of the examination via telemedicine. Fourth, users were very satisfied (49.4%) with the therapy and/or advice given via telemedicine. Finally, users were very satisfied (43.2%) with the privacy provided by telemedicine consultation.

## Discussion

Overall, as a predisposing factor, the COVID-19 pandemic affected ARD patients’ perspectives about their concerns about infection and promoted their preventive behaviors. This is aligned with the health belief model that shows that factors that contribute to patient’s perceptions can guide their behavior. Moreover, the COVID-19 situation and lockdown policy was an enabling factor and reinforcing factor for patients in health-seeking behavior. These behavioral changes obviously needed an intervention, and we showed that patients’ reception of telemedicine as an alternative health care practice during the pandemic was positively received.

In 2023, the Indonesian government ended COVID-19 restrictions and declared COVID-19 an endemic disease, which created another shift toward a new normal situation. However, some patients still faced treatment interruptions even before and after the pandemic due to Indonesia’s sociodemographic and geographical factors. Finally, from this pandemic, we learned that telemedicine could be a breakthrough for patients to access health care, especially for ARD patients who face barriers to access.

### Fear of COVID-19

The American College of Rheumatology (ACR) and APLAR declared that patients with ARDs are at risk and more susceptible to COVID-19 infection than the general population. However, it is still debated whether ARD medications may become a possible risk factor for ARD patients having poor outcomes, including hospitalization, severe infection, mortality, intensive care unit admission, and ventilator use in COVID-19. On the other hand, ARD medication should not be immediately stopped and should be maintained at a specific dose to prevent disease flares, and the prescription should be determined on an individualized basis according to the disease [23–25].

As seen in this study, this mindset may affect patient concerns about being infected and drive avoidance behavior toward COVID-19. A similar phenomenon has been reported in previous studies: in the study by Fragoulis et al., patients who changed medication due to concerns about the immunosuppression effect increased their susceptibility to infection and worse outcomes; [26] in the study by Michaud et al., patients added and removed their medication due to worries about COVID-19 infection; [27] in the study by Pineda-Sic et al., patients in Latin America changed medications due to fear of contracting COVID-19; [28] and in the study by Khabbazi et al., nonadherent patients in East Azerbaijan feared the immunosuppressive effect of medications [29]. Thus, in the future, patients need an understanding of COVID-19 infection, the importance of taking medication, and routine follow-up checks. The continuity of communication, information, and education between doctor and patient are key in managing therapy adherence to prevent a worse outcome.

Additionally, our study showed that sex was associated with stopping medication without medical advice. This may be because we had more female respondents than male respondents, and this might have been a selection bias during our analysis. In contrast, a previous study showed no significant difference in adherence to medication between sexes during the pandemic. However, the study examined nonspecific diseases, while our study examined rheumatic autoimmune patients who were predominantly females [30].

### Lockdown and social distancing policies during the COVID-19 pandemic

During the second wave of COVID-19 (June to August 2021), the Indonesian government implemented PPKM. PPKM tightened social distancing, including community activities and travel regulations. According to Instructions of the Ministry of Home Affairs number 27 (for Java and Bali) and 28 (for outside Java and Bali region), [2, 3] each region was classified into levels based on epidemiological indicators, surveillance indicators, and health care services. The majority of our respondents were living in level 3 and 4 zones, which were classified as high-risk areas at the time. However, there might have been recall bias as they filled out the questionnaire to evaluate their experience after the second wave was over.

In addition, PPKM levels 3 and 4 implemented (1) online teaching for all levels of education; (2) 100% work-from-home (WFH) activities for all nonessential activities; (3) 50% work from an office (WFO) for essential activities such as the economic sector and data centers; and (4) 100% WFO with strict health protocols for critical sectors such as health care facilities, disaster response teams, and governmental vital objects [2, 3]. The difference was in the maximum capacity of transportation, which was up to 50% at level 4 and up to 70% at level 3. However, in both level 3 and 4 zones, long-distance travelers required a vaccination card, polymerase chain reaction (PCR) test or rapid diagnostic test (RDT) for COVID-19 [2, 3].

Although there was no regulation inhibiting citizens from traveling for medical or emergency purposes, travel restrictions still became a barrier to access. A possible reason was that a COVID-19 certificate was one of the requirements needed for traveling across the region; however, it is possible that some ARD patients were not eligible to be vaccinated due to their disease activity and treatment, as evidenced by a letter from a rheumatologist. Moreover, PCR or RDT tests became an economical burden when patients needed to frequently travel for hospital visits. In particular, this regulation was implemented for long-distance travelers; however, most rheumatology centers are located in large cities, and some patients may have been referred from a distant hospital. In addition, patients were anxious about COVID-19 exposure while traveling. This becomes more evident in several studies conducted in Indonesia, there was a considerable decrease in the number of outpatient clinics other than ARD clinics during the lockdown, and a rebound was seen afterward due to these factors [31–34].

This problem was also evident in our study, in which blocked access during PPKM was associated with the need to avoid hospital visits. This showed that travel restrictions and transportation regulations were factors for patients to consider the use of telemedicine, especially during the pandemic. On the other hand, telemedicine had the potential role of preventing COVID-19 spread during travel; it also connected patients who were not able to travel to the health care facility, and most importantly, the patient had access to receive information from the health care worker about their medical condition and COVID-19 [35].

### Telemedicine in rheumatology Practice

In our study, respondents were very satisfied with telemedicine (3.8 ± 0.7 over 5), and more than half preferred telemedicine visits during the pandemic, but less than half preferred telemedicine visits after the pandemic. Similar results were evidenced in surveys by Tornero-Molina et al. in Spain, where patients showed higher overall satisfaction in tele-rheumatology (RTC) and scored 8.62 out of 10, and approximately 391/469 (84%) wanted to repeat RTC; [21] in the study by Jones et al., in most UK rheumatology outpatient clinics, 239/297 (84%) patients were satisfied with their health assessment, and 60% wanted to have routine follow-up telephone consultations; [36] in the study by Mortazavi et al. in the US, most of their rheumatology clinic’s patients (74%) were satisfied with their virtual visit; [37] and in the study by Cliffe and Stevenson in the UK, the majority of their musculoskeletal patients (194/241 (80.5%)) were delighted with a virtual consultation [38].

Alternatively, early in this pandemic, telemedicine was adopted into the Indonesian health care system and regulated by the Indonesian Ministry of Health and Indonesian Medical Council to tackle geographical and distance barriers between health care providers and patients [15, 39]. Indonesian national health insurance covered all medical bills with a well-documented medical record. Many commercial telemedicine providers could also be accessed on smart devices linked to private insurance. Nevertheless, most of our respondents, (126 (54.8%)) who had never used this service, did not know about telemedicine.

In early 2022, APLAR released a recommendation on telemedicine practice in rheumatology. They recommended telemedicine for situations in which rheumatologists and/or patients have a communication gap or when there is a disruption in regular health services to prevent unsupervised medication. Telemedicine should be based on clinical effectiveness, safety issues, the patient’s perspective, economic, organizational, sociocultural, ethical, and legal aspects, and equitable health access according to local regulations. Patient data privacy, integrity, and security should be protected. The decision should be shared by rheumatologists and patients and should be made after the preconsultation triage system to assess whether the patient’s condition is suitable for telemedicine follow-up. However, telemedicine is not recommended for an initial appointment or a patient with an unconfirmed diagnosis. Telemedicine is also recommended to train nurses, physicians, and rheumatologists to provide better clinical practice in telemedicine [14].

### Postpandemic situations

According to the Indonesian government, a rural area is defined by topography, access to urban facilities, agriculture, landscapes, or population density. Some rural areas are locations outside Java-Bali, which are the less developed areas, whereas others are classified according to population density and medical workforce supply [39]. However, in our study, there was no significant difference in health care disruption or treatment interruption between Java-Bali and outside the Java-Bali region.

In a study by Putri et al., [40] there was an inequality in specialist distribution across Indonesia based on geography. Doctors working in less developed areas may face a lack of health infrastructure and accommodation, even though the government has created policies and programs to provide financial and non-financial benefits to tackle this problem [40, 41]. These are probably the reasons that advanced health care practices such as rheumatology centers are concentrated in large cities. On the other hand, not all patients can accommodate travel to access health care.

Indeed, ARDs are chronic diseases that need to be monitored continuously and telemedicine may helps to overcome the shortage of rheumatologists or internists to provide care outside rheumatology centers. This helps patients who are unable to travel and prevents the discontinuation of medical follow-up [42]. Despite this limitation, there should be a new normal situation in rheumatology practice in Indonesia to meet the patient’s needs as we learned from the pandemic.

### Limitations

Despite the results, this study has several limitations. First, this was a cross-sectional study that could not explain causal or effective relationships. Second, the self-developed questionnaire might have led to information bias among the respondents. Third, respondents’ medical histories were not traced from their medical records, which might have led to selection bias. Because we do not have a national database for rheumatology patients. Last, ARD patients who completed the questionnaire might have been more concerned about COVID-19 and their diseases compared to those who did not. For example, our respondents are predominantly females, joining the autoimmune community, and those who filled out the questionnaire given by their internist or rheumatologist must have received more information about COVID-19 and the ARD than those who did not. In addition, respondents completed the questionnaire during the nonpeak of COVID-19 cases, which may have led to recall bias, even though the questionnaire was designed to evaluate the overall patient experience throughout the pandemic.

## Conclusions

In short, internal and external factors affected health care disruptions and treatment interruptions during the COVID-19 pandemic, which affected treatment adherence among ARD patients. Moreover, during the pandemic, the use of telemedicine was significantly associated with respondents who avoided hospital visits, and users showed high satisfaction with telemedicine services. Thus, in the future, telemedicine may become an alternative in rheumatology practice in Indonesia to increase visitation and treatment adherence, which is interesting because Indonesia’s sociodemography and geographical situation are the biggest challenges in health care practice.

## Electronic supplementary material

Below is the link to the electronic supplementary material.

Supplementary Material 1

Supplementary Material 2

## Data Availability

The datasets generated and/or analyzed during the current study are available in the Mendeley repository (https://data.mendeley.com/datasets/s7cghps827/1).
