# Supplementary material for: Learning from the COVID-19 pandemic: health care disturbances and telemedicine as an alternative rheumatology practice in Indonesia
Source: BMC Health Serv Res. 2023 May 8;23:451. doi: 10.1186/s12913-023-09389-5 (PMC10165285; doi:10.1186/s12913-023-09389-5)
Supplement: Supplementary file 1 — Supplementary Material 1 [file 12913_2023_9389_MOESM1_ESM.pdf]

INFORMED CONSENT
(online survey)

Dear. Ladies and gentlemen, brothers and sisters

I, Dr. dr. Rudy Hidayat, SpPD-KR, FINASIM from the Department of Internal Medicine FKUI-RSCM will conduct a research entitled Perception, Behavior, and External Factors Affecting Health Services and Use of Telemedicine in Autoimmune Rheumatic Disease Patients during the COVID-19 Pandemic. The data that will be generated from the research is a reference to determine perceptions, behaviors, and external factors as well as the level of patient satisfaction with the use of telemedicine during the COVID-19 pandemic. All data you provide is confidential, stored in the researcher's e-mail, and can only be accessed by the research team.

For this reason, we hope that you are willing to participate in this research by answering the questions in this questionnaire which consists of 38 questions regarding perceptions, behaviors, and external factors that cause obstacles to health services during the COVID-19 pandemic. . This charging will take about 5-10 minutes of your time. Your participation in this research is voluntary. You do not have to participate in this research if you do not wish to. Your participation will be kept confidential and you will/will not be contacted again in the future. No specific incentive or reward will be given to you for participating in this research. This research involves no foreseeable risk to you and no direct benefit. However, the benefit of your participation can be t is to develop public health education and are policies during the COVID-19 pandemic.

If you are willing to participate in this research, please fill in and put a ☑ mark on the statement 'Agree to participate in this research' below. If you have further questions about this research or if you have any research-related concerns, you may contact dr. Faisal Parlindungan, SpPD-KR at faisal.parlindungan@alumni.ui.ac.id.

For your attention, we thank you.

Yours faithfully,
Dr. dr. Rudy Hidayat, SpPD-KR, FINASIM

I have read all the descriptions of this research. I, who have identified below knowingly and without coercion, are willing to participate in this research. I guarantee that all data and information that I provide in this research are honest and true.

- ☐ Agree to participate in this research
- ☐ Disagree to participate in this research

QUESTIONNAIRE

“Perception, Behavior and External Factors Affecting Health Services and Use of Telemedicine in Patients with Autoimmune Rheumatic Disease during the COVID-19 Pandemic”

PART 1: Identity

1. Name : (Initial)
2. Date of Birth : dd/mm/yyyy
3. Gender : M/F
4. Occupation :
5. Education` :
6. Domicile :

PART 2: Basic Medical Data

1. What diagnosis of autoimmune rheumatic disease did your doctor tell you? (Can choose more than one answer)Artritis Reumatiod/RA
    a. Systemic Lupus Erythematosus (SLE)
    b. Psoriatic Arthritis
    c. Ankylosing Spondylitis
    d. Sjogren Syndrome
    e. Scleroderma/ Systemic Sclerosis
    f. Other: …
2. Medicines for autoimmune rheumatic diseases: (Can choose more than one answer)
    a. Not receiving treatment
    b. Metotrexate (Rheutrex, dll)
    c. Cyclophosphamid
    d. Cyclosporin (Sandimun)
    e. Azatioprin (Imuran)
    f. Natrium mikofenolat (Myfortic)
    g. Mofetil Mikofenolat (Cellcept/Kamyfet/ Myrept)
    h. Leflunomid (Arafa)
    i. Sulfasalazine (Sulcolon/Lazafin/ Sulfitis)
    j. Rituximab (Mabthera)
    k. IL-6 Tocilizumab, (Actemra)
    l. Anti-IL17 (Fraizeron)
    m. Anti TNF (Enbrel/Humira/ Simponi/Remicade)
    n. Prednison
    o. Methylprednisolon
    p. Prednisolon

3. During the COVID-19 pandemic, are you still doing your daily activities or working as usual?
    a. Yes
    b. No
4. Have you ever been infected with COVID-19?
    a. Yes
    b. No

PART 3: Perception, Behavior, External Factors Affecting Health Services during the COVID-19 Pandemic

Fill in this questionnaire by ticking (V) in the column that has been provided according to your condition.

Information :
1 = Never
2 = Rarely
3 = Sometimes
4 = Often
5 = Always

No	Questions	1	2	3	4	5
	Concern					
1	Are you worried about COVID-19 infection?					
2	Are you concerned that autoimmune rheumatic diseases may increase your risk of COVID-19 infection?					
3	Are you worried that being infected with COVID-19 will increase the risk of worsening symptoms of autoimmune rheumatic diseases?					
4	Are you worried that if you are infected with COVID-19, you will get more severe COVID-19 symptoms than COVID-19 patients without rheumatic-autoimmune disease?					
	Behavior					
5	Do you avoid going out of the house?					
6	Do you avoid going grocery shopping?					
7	Do you avoid activities that require physical encounters with friends/family/coworkers?					

External Factors

5. Is access to the hospital where you seek treatment blocked during the PPKM?
    a. Yes
    b. No
6. What transportation do you use to go to the hospital?
    a. Private vehicle
    b. Public transportation

PART 4: Respondents' Satisfaction with Telemedicine Services

1. Have you ever consulted via telemedicine?
   a. Once
   b. Never, please state why… (Continue to Part 5: Closing Questions)
2. What type of meeting via telemedicine do you prefer?
   a. Consultation by text/chat via commercial applications or hospital telemedicine service
   b. Video-consultation with doctors via commercial applications or hospital telemedicine service
   c. Make an appointment for a face-to-face consultation
   d. Others, mention…

Satisfaction with Telemedicine Services

Fill in this questionnaire by ticking (V) in the column that has been provided according to your condition.
Information :
1 = Very dissatisfied
2 = Not satisfied
3 = Neutral
4 = Satisfied
5 = Very satisfied

No	Questions	1	2	3	4	5
3	How satisfied are you with the convenience of consulting via telemedicine?					
4	How satisfied are you with the ease of access to consultation via telemedicine?					
5	How satisfied are you with the accuracy of the examination via telemedicine?					
6	How satisfied are you with the therapy and/or advice provided through telemedicine?					
7	How satisfied are you with the security of your privacy and personal data when you consult via telemedicine?					

PART 5: Final Question

1. Do you feel the need to avoid going to the hospital?
   a. Yes
   b. No
2. Do you feel the need to avoid going to other clinical/diagnostic laboratories to perform other laboratory tests or diagnostics?
   a. Yes
   b. No
3. Have you ever tried to stop medication without the direction of the treating doctor during the COVID-19 pandemic?
   a. Yes
   b. No
4. Have you ever tried to continue treatment on your own without the direction of the treating doctor during the COVID-19 pandemic because you didn't control it in time?

a. Yes
b. No
