# Supplementary material for: Learning from the COVID-19 pandemic: health care disturbances and telemedicine as an alternative rheumatology practice in Indonesia
Source: BMC Health Serv Res. 2023 May 8;23:451. doi: 10.1186/s12913-023-09389-5 (PMC10165285; doi:10.1186/s12913-023-09389-5)
Supplement: Supplementary file 2 — Supplementary Material 2 [file 12913_2023_9389_MOESM2_ESM.docx]

**STROBE Statement—checklist of items that should be included in reports of observational studies**

|  | Item No. | Recommendation | Page  No. | Relevant text from manuscript |
| --- | --- | --- | --- | --- |
| **Title and abstract** | 1 | (*a*) Indicate the study’s design with a commonly used term in the title or the abstract | 1 | A Cross-sectional Study |
|  |  | (*b*) Provide in the abstract an informative and balanced summary of what was done and what was found | 2 | Abstract |
| Introduction | | | |  |
| Background/rationale | 2 | Explain the scientific background and rationale for the investigation being reported | 3 | Introduction – Paragraph 1-3 |
| Objectives | 3 | State specific objectives, including any prespecified hypotheses | 3 | Introduction – Paragraph 3-4 |
| Methods | | | |  |
| Study design | 4 | Present key elements of study design early in the paper | 3 | Materials and Methods – Study Population |
| Setting | 5 | Describe the setting, locations, and relevant dates, including periods of recruitment, exposure, follow-up, and data collection | 3 | Materials and Methods – Study Population |
| Participants | 6 | 1. ***Cohort study***—Give the eligibility criteria, and the sources and methods of selection of participants. Describe methods of follow-up 2. ***Case-control study***—Give the eligibility criteria, and the sources and methods of case ascertainment and control selection. Give the rationale for the choice of cases and controls 3. ***Cross-sectional study***—Give the eligibility criteria, and the sources and methods of selection of participants | 3 | Materials and Methods – Study Population |
|  |  | 1. ***Cohort study***—For matched studies, give matching criteria and number of exposed and unexposed 2. ***Case-control study***—For matched studies, give matching criteria and the number of controls per case |  | Not applicable |
| Variables | 7 | Clearly define all outcomes, exposures, predictors, potential confounders, and effect modifiers. Give diagnostic criteria, if applicable | 3-4 | Materials and Methods |
| Data sources/ measurement | 8* | For each variable of interest, give sources of data and details of methods of assessment (measurement). Describe comparability of assessment methods if there is more than one group | 3-4 | Materials and Methods |
| Bias | 9 | Describe any efforts to address potential sources of bias | 8 | Discussion – Limitation (Last Paragraph) |
| Study size | 10 | Explain how the study size was arrived at | 3 | Materials and Methods – Study Population (Samples was derived consecutively from outpatient clinic) |

| Quantitative variables | | 11 | | Explain how quantitative variables were handled in the analyses. If applicable, describe which groupings were chosen and why | 4 | Materials and Methods (DAS28-CRP, HADS, Cytokine Analysis) |
| --- | --- | --- | --- | --- | --- | --- |
| Statistical methods | | 12 | | (*a*) Describe all statistical methods, including those used to control for confounding | 4 | Materials and Methods – Statistical Analysis |
|  |  |  |  | (*b*) Describe any methods used to examine subgroups and interactions | 4 | Materials and Methods – Statistical Analysis |
|  |  |  |  | (*c*) Explain how missing data were addressed |  | Not required, no missing data |
|  |  |  |  | (*d*) *Cohort study*—If applicable, explain how loss to follow-up was addressed  *Case-control study*—If applicable, explain how matching of cases and controls was addressed  *Cross-sectional study*—If applicable, describe analytical methods taking account of sampling strategy | 4 | Results – Paragraph 1 |
|  |  |  |  | (*e*) Describe any sensitivity analyses |  | Not applicable |
| Results | | | | | | |
| Participants | | 13* | | (a) Report numbers of individuals at each stage of study—eg numbers potentially eligible, examined for eligibility, confirmed eligible, included in the study, completing follow-up, and analysed | 4 | Results – Paragraph 1 |
|  |  |  |  | (b) Give reasons for non-participation at each stage |  | Not applicable |
|  |  |  |  | (c) Consider use of a flow diagram |  | Not applicable |
| Descriptive data | | 14* | | (a) Give characteristics of study participants (eg demographic, clinical, social) and information on exposures and potential confounders | 4 | Results – Paragraph 1, Table 1 |
|  |  |  |  | (b) Indicate number of participants with missing data for each variable of interest |  | Not required, no missing data |
|  |  |  |  | (c) *Cohort study*—Summarise follow-up time (eg, average and total amount) |  |  |
| Outcome data | | 15* | | *Cohort study*—Report numbers of outcome events or summary measures over time |  |  |
|  |  |  |  | *Case-control study—*Report numbers in each exposure category, or summary measures of exposure |  |  |
|  |  |  |  | *Cross-sectional study—*Report numbers of outcome events or summary measures | 4 | Results – Paragraph 1, Table 1 |
| Main results | | 16 | | (*a*) Give unadjusted estimates and, if applicable, confounder-adjusted estimates and their precision (eg, 95% confidence interval). Make clear which confounders were adjusted for and why they were included | 4-5 | Results – Paragraph 1-5 , Table 1-4 |
|  |  |  |  | (*b*) Report category boundaries when continuous variables were categorized | 4-5 | Results – Paragraph 1-5 , Table 1-4 |
|  |  |  |  | (*c*) If relevant, consider translating estimates of relative risk into absolute risk for a meaningful time period |  | Not required |
| Other analyses | 17 | | Report other analyses done—eg analyses of subgroups and interactions, and sensitivity analyses | | 4-5 | Results – Paragraph 1-5 , Table 2-4 |
| Discussion | | | | | | |
| Key results | 18 | | Summarise key results with reference to study objectives | | 5-8 | Discussions |
| Limitations | 19 | | Discuss limitations of the study, taking into account sources of potential bias or imprecision. Discuss both direction and magnitude of any potential bias | | 8 | Discussion – Limitation (Last Paragraph) |
| Interpretation | 20 | | Give a cautious overall interpretation of results considering objectives, limitations, multiplicity of analyses, results from similar studies, and other relevant evidence | | 5-8 | Discussions |
| Generalisability | 21 | | Discuss the generalisability (external validity) of the study results | | 7 | Discussions |
| Other information | | |  | | | |
| Funding | 22 | | Give the source of funding and the role of the funders for the present study and, if applicable, for the original study on which the present article is based | | 8 | Funding |

***Give information separately for cases and controls in case-control studies and, if applicable, for exposed and unexposed groups in cohort and cross-sectional studies.**
